# Gene Editing in Rabbits: Unique Opportunities for Translational Biomedical Research

**DOI:** 10.3389/fgene.2021.642444

**Published:** 2021-01-28

**Authors:** Jie Xu, Jifeng Zhang, Dongshan Yang, Jun Song, Brooke Pallas, Chen Zhang, Jiafen Hu, Xuwen Peng, Neil D. Christensen, Renzhi Han, Y. Eugene Chen

**Affiliations:** ^1^Center for Advanced Models for Translational Sciences and Therapeutics, University of Michigan Medical Center, University of Michigan Medical School, Ann Arbor, MI, United States; ^2^Unit for Laboratory Animal Medicine, University of Michigan Medical School, Ann Arbor, MI, United States; ^3^Biomedical Sciences and Biophysics Graduate Program, Division of Cardiac Surgery, Department of Surgery, Dorothy M. Davis Heart and Lung Research Institute, The Ohio State University Wexner Medical Center, Columbus, OH, United States; ^4^Department of Pathology and Laboratory Medicine, Penn State Cancer Institute, Hershey, PA, United States; ^5^Department of Comparative Medicine, Penn State University College of Medicine, Hershey, PA, United States; ^6^Department of Microbiology and Immunology, Penn State University College of Medicine, Hershey, PA, United States

**Keywords:** gene editing, Cas9, rabbit models, biomedical research, human diseases

## Abstract

The rabbit is a classic animal model for biomedical research, but the production of gene targeted transgenic rabbits had been extremely challenging until the recent advent of gene editing tools. More than fifty gene knockout or knock-in rabbit models have been reported in the past decade. Gene edited (GE) rabbit models, compared to their counterpart mouse models, may offer unique opportunities in translational biomedical research attributed primarily to their relatively large size and long lifespan. More importantly, GE rabbit models have been found to mimic several disease pathologies better than their mouse counterparts particularly in fields focused on genetically inherited diseases, cardiovascular diseases, ocular diseases, and others. In this review we present selected examples of research areas where GE rabbit models are expected to make immediate contributions to the understanding of the pathophysiology of human disease, and support the development of novel therapeutics.

## Introduction

The rabbit (*Oryctolagus cuniculus*) is a classic research animal model. For example, in the nineteenth century, it was used to develop the rabies vaccine by Louis Pasteur (Esteves et al., 2018). Since the early 1900s, the rabbit has been the preferred species for polyclonal antibody (pAb) production, and today, is responsible for over 40% of the total pAb used in research labs and medical clinics (CiteAb., 2015). In the late 1970s, rabbit models provided insights into the molecular and cellular mechanisms of atherosclerosis and contributed to the development of Statins, the most potent class of lipid-lowering drugs prescribed annually for millions of patients worldwide (Shiomi, 2020).

Yet the contributions of this model species to biomedical research have been outshined by mouse models since the 1980s, primarily due to the following two factors. First, the development of germline transmitting embryonic stem cells (ESCs) allows for targeted genetic manipulations (GM) including gene knockout, precision mutation, and many other versatile modifications to be efficiently implemented in the mouse genome (Evans and Kaufman, 1981; Martin, 1981; Gossler et al., 1986; Robertson et al., 1986). Unfortunately, despite decades of efforts, no group has been able to develop germline transmitting ESCs in rabbits or any other non-rodent mammalian species. This lack of ability to manipulate at the genetic level and the consequent failure in producing GM rabbits stood as a major hurdle to their application in modern biomedical research.

Another practical factor limiting the availability of rabbit models is the economic affordability. The mice are of small size (adult 20–35 g), produce large litters (2–12 pups/litter), have a short gestational period (19–21 days), and quickly reach sexual maturation (6–8 weeks). These traits allow researchers to easily house large number of mice and to quickly expand the colony for their specific research needs. In contrast, while rabbits produce a similar litter size of 4–12 kits/litter and possess a comparable gestational length of 4 weeks, they on average weigh 3–4 kg as adults (New Zealand White strain), and require ∼5–6 months to reach sexual maturation, making one reproductive generation over two times longer than in mice. Also, being over 100 times more massive than a mouse, commonly translates at most research institutes into a rabbit housing *per diem* rate that is greater than 10 times that of a mouse. Given these facts, it is not surprising that mice represent >70% of all mammalian animal models utilized in the United Kingdom; whereas the use of rabbit models is merely 1% that of mouse models (National Statistics, 2019). We argue that to some extent, this relative dominance of mouse models more reflects a “use what is available and affordable” approach, rather than a “use what is physiologically accurate and translational” approach, for any given biomedical question.

**FIGURE 1:**
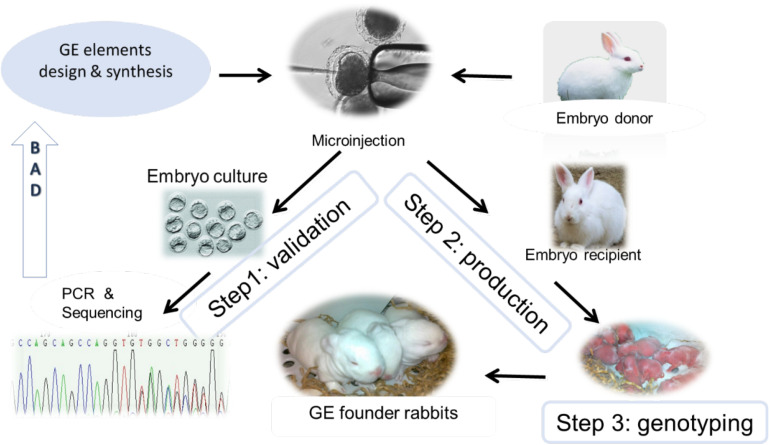
Illustration of GE rabbit production. After GE element design and synthesis, they will be subjected to *in vitro* validation (Step 1) using embryos harvested from embryo donor animals. In the event of Step 1 failure (“BAD” arrow), GE elements will be re-designed and validated. After passing Step 1 validation, GE elements will be microinjected to pronuclear stage embryos followed by embryo transfer to an embryo recipient female rabbit (Step 2). After the one-month gestation time, newborn kits will be genotyped, and confirmed GE founder rabbits will be kept for herd expansion (Step 3).

The advent of gene editing nucleases, first Zinc Finger Nuclease (ZFN), next Transcription Activator-Like Effector Nuclease (TALEN), and most recently the Nobel Prize winning technology Clustered Regularly Interspaced Short Palindromic Repeats/CRISPR-associated protein-9 (CRISPR/Cas9) have brought a revolution to animal model development (Jacinto et al., 2020). These engineered endonucleases are efficient in generating double-strand breaks (DSB) in specific genomic loci that are repaired either by error-prone non-homologous end joining (NHEJ), resulting in a functional knockout of the targeted gene or through homologous recombination (HR) which is exploited to integrate a designed DNA sequence at a specific locus. Thanks to the very high targeted editing efficiencies, gene knockout and knock-in animals can be now readily derived by injection of gene editing elements (e.g., Cas9 protein or its coding DNA, mRNA, guide RNA, and donor DNA) into pronuclear stage embryos followed by embryo transfer, bypassing the need for germline transmitting stem cells (Figure 1).

**FIGURE 2:**
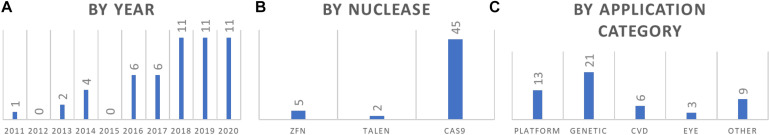
Summary of GE rabbit publications. **(A)** Summary of reports by year. **(B)** Summary of reports by nuclease that was used. **(C)** Summary by application categories of the models.

More than 50 gene knockout and knock-in rabbit models (Table 1) have been produced using ZFN, TALEN, and Cas9 in the past decade, according to a PUBMED search conducted on December 7, 2020 (Figure 2 and Supplementary Table 1). A comprehensive review of gene editing in rabbits with a focus on the methodology is published elsewhere (Yang et al., 2019). Readers can refer to that review to gain knowledge on how to harness these powerful tools for generating a gene edited (GE) rabbit model. Briefly, gene editing nuclease elements are designed and synthesized for generating the targeted genetic changes in rabbits, *in vitro* validated, followed by embryo transfer and genotyping of the founders (Figure 1). Confirmed founder animals are then used for breeding to expand the herd.

**TABLE 1:** Comparison of selected parameters of cardiovascular physiology between mouse, rabbit, and human.

	Mouse	Rabbit	Human
Cardiac sarcomere composition	α- myosin heavy chain (MHC)	β-MHC	β-MHC
Lipoprotein profile	HDL-rich	LDL-rich	LDL-rich
CETP	No	Yes	Yes
Dietary cholesterol	Resistant	Sensitive	Sensitive
Atherosclerosis	Resistant	Susceptible	Susceptible

Among the 52 reports of GE rabbits, some interesting patterns are revealed (Figure 2). First, more than half of these models (33/52) are reported in the past 3 years (2018–2020), indicating an upward trend of using GE rabbits in biomedical research. Second, CRISPR/Cas is the dominant nuclease of choice, accounting for the production of more than 85% (45/52) of these models. In terms of application categories, while a quarter of these reports are about the development and optimization of the GE platform (13/52), the others are mostly for developing models in biomedical research, with the most for genetic diseases (40%, 21/52), followed by CVD (12%, 6/52) and eye diseases (6%, 3/52).

Several general features of the rabbit make it often a preferred model species in biomedical applications (Honda and Ogura, 2017; Fan et al., 2018). Unlike pigs and non-human primates, rabbits are relatively inexpensive and can be easily adapted to existing research facility infrastructures. They are easy to breed and handle and are recognized by the scientific and regulatory communities as a well-established laboratory friendly model species. Compared to rodents, rabbits are phylogenetically closer to humans (Li et al., 1990; Graur et al., 1996). Also, the relatively large size of rabbits makes it much easier to perform surgical procedures, collect serial blood samples, and perform tissue and organ biopsies than in a mouse. For example, the abdominal and thoracic aorta of an adult rabbit is ∼3 mm, comparable to that of an adult human coronary artery (Dodge et al., 1992); whereas the aorta diameter of a mouse is smaller than 1 mm. Hence it is more practical and translational to conduct blood vessel transplantation in a rabbit than in a mouse, which is necessary for preclinical evaluation of stem-cell derived or other types of synthetic blood vessels. The relatively longer lifespan also matters in certain applications. For example, to test both the therapeutic efficacy and safety profile of viral based therapies, such as adeno associated virus (AAV) mediated gene editing therapy, it is preferred to monitor safety profiles in a preclinical model system over a course of several years before it is entered into human clinical trials. In this regard, rabbit models could represent a viable option.

Historically, the rabbit has served as classic models for the studies of cardiovascular diseases (CVD), infectious diseases and eye diseases. In recent years, thanks to the completion of rabbit genome project and the availability of gene editing nucleases, it has been increasingly recognized that the rabbits may serve as clinically relevant models for other human diseases including many genetic diseases. Here we present a few examples to demonstrate the perceived advantages of GE rabbits in translational biomedical research.

## Gene Edited Rabbits for Translational Studies of Cardiovascular Diseases

There is ample evidence to support that rabbits are appropriate animal models for the study of human CVD (Table 1) primarily due to the similarities between humans and rabbits in lipid metabolism, blood lipoprotein profiles, and their physiological responses to the western diet (Fan et al., 2015, 2018; Zhang et al., 2017). As in humans, the apoB-containing particles (very-low-density lipoprotein, VLDL and low-density lipoprotein, LDL) are the major lipoproteins in rabbits carrying cholesterol in the blood, especially when fed a cholesterol-rich diet. Rabbits, but not mice, possess cholesteryl ester transfer protein (CETP), a critical regulator of HDL levels and cholesterol metabolism in humans. Additionally, wild-type rabbits are easily induced into a hyperlipidemic state when fed a cholesterol-rich diet and rapidly develop aortic and coronary artery atherosclerosis. Therefore, wild-type and transgenic rabbits are widely used for studying human lipid metabolism and related CVD, including atherosclerosis. Since 2009, the emergence of genome editing technologies, including ZFN, TALEN, and CRISPR/Cas9 described earlier, has made it possible to generate GE rabbits for the study of human diseases. In the last decade, many gene knockout and knock-in rabbits were produced by these tools for human CVD related research. Here are some examples.

In humans, plasma HDL levels are inversely correlated with the development of atherosclerosis and myocardial infarction (Inazu et al., 1990). With the first generation gene-editing tool, ZFN, Zhang et al. (2017) generated CETP knockout rabbits. As expected, the CETP knockout rabbits showed higher HDL levels than the wild-type controls under either standard diet or cholesterol-rich diet fed conditions. Furthermore, the HDL isolated from CETP KO rabbits exhibited increased cholesterol efflux capacity from cholesterol-loaded macrophages than the HDL from wild-type rabbits. The CETP knockout rabbits also showed reduced total cholesterol (TC) levels, mainly contributed by the decreased apoB containing particles. Finally, the CETP knockout rabbits are protected against cholesterol-rich diet-induced aortic and coronary atherosclerosis, likely because of lower β-VLDL and higher HDL levels and the HDL functionality. These results support the need for continuing efforts to develop novel CETP inhibitors for the treatment of hypercholesterolemia and atherosclerosis.

It is well established that high triglyceride (TG) levels in the blood is an independent risk factor for overall CVD (Reiner, 2017). ApoCIII mediates the metabolism of triglyceride-rich lipoproteins. To further delineate the role of apoCIII in lipoprotein metabolism, Yan et al. (2020) generated apoCIII knockout rabbits using the ZFN technique. The apoCIII knockout rabbits showed a faster TG clearance rate with significantly lower plasma TG levels, accompanied by an apparent reduction of VLDL and intermediate-density lipoprotein (IDL). The apoCIII heterozygous and homozygous knockout rabbits exhibited significantly lower levels of plasma TC and TG when fed a cholesterol-rich diet and reduced atherosclerosis in both aortic and coronary arteries, when compared to wild-type control rabbits. These results support the concept that apoCIII is a promising drug target for treating hypertriglyceridemia.

ApoAII is the second major apolipoprotein of the HDL particle. However, the physiological functions of apoAII are not fully understood (Koike et al., 2009; Wang et al., 2013). Rabbits are unique in that they naturally lack the apoAII gene. By utilizing TALEN, Koike et al. (2020) generated the apoAII knock-in rabbit by inserting the human apoAII coding sequence into the rabbit apoAI gene locus. This human apoAII knock-in rabbit can be an animal model to study the specific functions of apoAII, independent of apoAI. ApoAII knock-in rabbits showed increased HDL levels and decreased TG levels due to accelerated clearance of TG-rich lipoproteins and higher lipoprotein lipase activity than the wild-type littermates. When fed a cholesterol-rich diet, apoAII knock-in rabbits were resistant to diet-induced hypertriglyceridemia and developed significantly less aortic atherosclerosis than wild type rabbits. These results suggest that apoAII containing HDL may have the potential to treat patients with hypertriglyceridemia and atherosclerosis.

The apoE knockout mouse is a widely used model for the study of human hyperlipidemia. With the CRISPR/Cas9 technology, we and others have generated the apoE knockout rabbits (Yang et al., 2014; Niimi et al., 2016; Yuan et al., 2019). The homozygous apoE knockout rabbits showed mild hyperlipidemia when fed a normal diet but developed much more severe hyperlipidemia when challenged with a cholesterol-rich diet, compared to the wild-type control rabbits. Consequently, the apoE knockout rabbits also developed more significant aortic atherosclerosis than wild-type rabbits upon cholesterol-rich diet challenge for as short as 10 weeks. With multiple sgRNAs targeting rabbit genes, Yuan et al. (2019) produced apoE and LDLR double knockout rabbits. This rabbit model can be used to investigate spontaneous hyperlipidemia and atherosclerosis. As the gene-editing tools are constantly improving, more and more precisely gene-edited rabbit models will be generated to improve our understanding of human CVD and develop novel therapeutic strategies for patients.

## Gene Edited Rabbits for Translational Studies of Human Papillomavirus

Human papillomavirus (HPV) is the most common sexually transmitted infection and is responsible for more than 90% of anal and cervical cancers, about 70% of vaginal and vulvar cancers, and 60% of penile cancers. To better understand host immune responses to papillomaviruses (PVs), effective preclinical models of natural infection and subsequent progression to malignancy are needed (Campo, 2002; Brandsma, 2005).

Rabbits have long served as the gold standard model for the study of PV pathogenesis, as well as its associated diseases and cancers (Cladel et al., 2019). Domestic rabbits are susceptible to infection by the cutaneous-tropic cottontail rabbit PV (CRPV) and the mucosal-tropic rabbit oral PV (ROPV) (Christensen et al., 2000a,b,c; Peh et al., 2002; Wilgenburg et al., 2005; Maglennon et al., 2011). These preclinical models have proven valuable for understanding the pathogenesis of high-risk papillomavirus infection and cancer development (Breitburd et al., 1997; Brandsma, 2005; Christensen et al., 2017), as well as played a pivotal role in the development of the two currently available prophylactic vaccines (Breitburd et al., 1995; Christensen et al., 1996; Cladel et al., 2019). In addition, the rabbit model has been used widely to test anti-viral and anti-tumor therapies (Christensen et al., 2000c, 2001, 2014). These rabbit models will continue to add to our understanding of mechanisms of viral pathogenesis, immunology and cancer development in the coming years.

**FIGURE 3:**
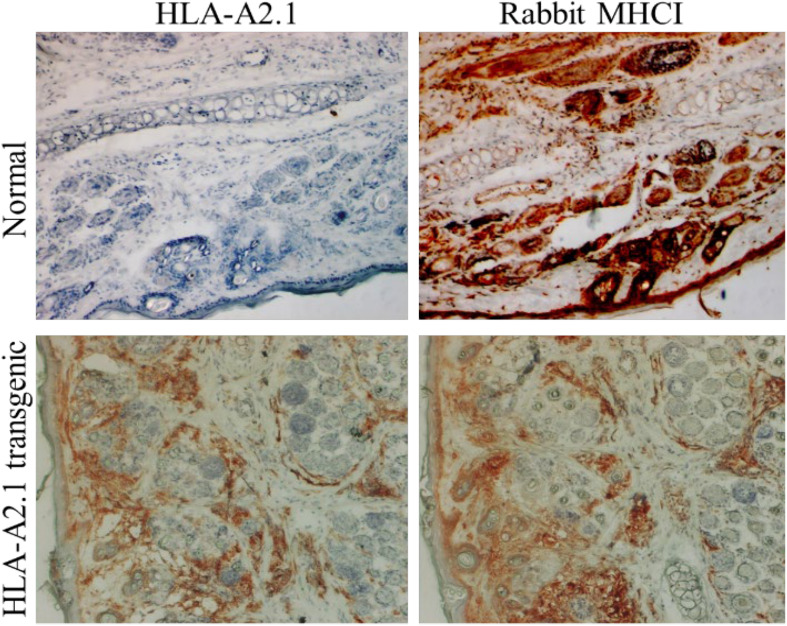
An HLA-A2.1 transgenic rabbit model. Ear biopsies from a normal rabbit and an HLA-A2.1 transgenic rabbit were stained for antibodies against either HLA-A2.1 (BB7.2 clone) or rabbit MHCI. The HLA-A2.1 transgenic rabbit (lower panels) expressed both HLA-A2.1 and rabbit MHCI on the cell surface while the wild-type rabbit (upper panels) showed no HLA-A2.1 expression but strong MHCI (20×). These rabbits have been used for studying HPV associated pathogenesis and T cell-based vaccine development against papillomavirus.

The Christensen, Hu and Peng’s research team at Penn State University Cancer Institute has been focusing on the development of effective preventive and therapeutic vaccines targeting host cell-mediated immunity in HPV using rabbit models (Han et al., 1999a,b; Hu et al., 2002, 2010a, 2014a). They have demonstrated that host immune responses, especially T cell mediated immune responses, play a key role in the disease outcome that was confirmed using an immune-suppressor such as cyclosporine A (CsA) (Hu et al., 2005; Osborne et al., 2020). To facilitate vaccine development against HPV, they established a novel human MHCI (HLA-A2.1) transgenic rabbit for studies on the role of CD8 T-cells in protective and therapeutic immunity (Hu et al., 2006; Figure 3). Computer prediction algorithms were employed to select, screen and identify several HLA-A2.1 restricted epitopes from rabbit PV as well as from HPV16 E6 and E7 (Hu et al., 2006, 2010b, 2014b; Bounds et al., 2011). Their studies demonstrated that this model system could be used to identify therapeutic targets for the control of HPV associated diseases (Hu et al., 2008). Specifically, several genes including APOBEC2 and IL36γ were found to be significantly dysregulated in persistent rabbit tumors, consistent with profiles in HPV-associated cancers. Given these findings, APOBEC2 and IL36γ gene knockout rabbits would greatly contribute to delineate whether these two genes play critical roles in PV-associated cancer progression. These GE rabbit models, once established, are expected to not only facilitate the integration of the CRPV rabbit model in human translational research, but also support the validation of new diagnostic and therapeutic targets for HPV cancer treatment.

## Gene Edited Rabbits for Translational Studies of AIDS

In an early review article, rabbits, chimpanzees and gibbons are listed as the only three animal species that were found susceptible to HIV-1 infection (Gardner and Luciw, 1989), which contrasts to the fact that rodents and many non-human primates do not get infected by HIV-1. Several molecular barriers, including members of the tripartite interaction motif (TRIM) family, members of the apolipoprotein B editing complex (APOBEC) class of cytidine deaminases, and CD317 (Tetherin, BST-2), are frequently up-regulated by host cells in response to viral (e.g., HIV-1) infection, and impose particularly effective barriers in the context of cross-species transmission of viruses (Brehm et al., 2014). Indeed, such human-specific infectivity makes the development of rodent AIDS models a great challenge (Hoang et al., 2008).

After decades of efforts, there are now mainly three approaches to establish mouse AIDS models: (i) humanize immunodeficient mice by xenotransplanting human tissues/cells; (ii) modify the virus; and (iii) genetically modify the animal genome. The first one dominates the current research community (Hoang et al., 2008; Zhang and Su, 2012; Brehm et al., 2014). The humanized mouse models include mice engrafted with human peripheral blood mononuclear cells (hu-PBL-SCID), mice surgically implanted with xenografts containing human fetal thymus and liver tissues (SCID-hu thy/liv), non-obese diabetic (NOD)/SCID mice surgically implanted with fetal bone marrow/liver/thymus (BLT), and others. It is noted that the development of these chimeric models is technically challenging, time-consuming, and not amenable to widespread use. The second approach modifies the viral genome so that it evades the species barriers upon entry and post entry; however, reagents that are effective on the modified virus may not work for the traditional HIV-1 virus. The third approach involves gene targeting in the animal genome to remove the species-specific molecular barriers (Tervo and Keppler, 2010); after removing all these barriers, it is expected that HIV-1 would efficiently infect and replicate in the animal. A number of these barriers such as CD4, CCR5, CXCR4, and cyclin T1, have been characterized and overcome in rodents. Nevertheless, this approach has been relatively unsuccessful in mice; the problems are largely due to ill-defined late-phase barriers that limit HIV production, particularly in primary T cells (Tervo and Keppler, 2010). As a result, mice and rats are still considered non-permissive to HIV infection.

The fact that rabbits are susceptible for HIV-1 infection suggests that this model species may be optimized to serve as an immunocompetent animal model of AIDS. It has been reported that expressing human CD4 and CCR5 are needed for efficient HIV-1 infection in rabbits (Hoang et al., 2008). Tervo and Keppler (2010) has generated hCD4/hCCR5 double transgenic rabbits, and reported that in hCD4/hCCR5 rabbit cells, envelope-specific and coreceptor-dependent entry of HIV-1 was achieved. However, HIV DNA synthesis was blocked in rabbit cells, likely mediated by TRIM5, which can be overcome by mutating the HIV-1 *gag* gene (Schaller et al., 2007; Tervo and Keppler, 2010). The authors showed that *gag*-modified HIV-1 produced by rabbit T cells was highly infectious, but those produced by macrophages have defects in infectivity. So while it is possible that the high permissivity of T cells in hCD4/hCCR5 transgenic rabbits alone may be sufficient for establishing a rabbit AIDS model, the requirement for altering the *gag* gene sequence to overcome the rabbit TRIM5 block would limit the utility of the model (Tervo and Keppler, 2010).

Tervo and Keppler et al. have delineated the molecular barriers in rabbits, in comparison with mice and rats during the HIV-1 replication cycle in primary cells (Table 2). Unlike mice and rats, primary cells from rabbits supported the functions of the regulatory viral proteins Tat and Rev, Gag processing, and the release of HIV-1 particles at levels comparable to those in human cells. It is important to point out that Tat-dependent LTR transactivation and Rev- and RRE-dependent viral gene expression are known barriers to HIV-1 replication in mice and rats, which are found absent in rabbits. Hence establishing a transgenic rabbit AIDS model is predicted to encounter much fewer molecular barriers. The authors then presented the two immediate molecular barriers to be targeted toward a GE rabbit model of AIDS: (i) the reverse transcription barrier mediated by rabbit TRIM5 and (ii) the macrophage-specific virion infectivity barrier. The latter is likely mediated by the rabbit ortholog of mammalian APOBEC1 protein, which has been shown to have anti-HIV activity (Ikeda et al., 2008). As such, it is of great interest in the GE rabbit community whether these suggested gene knockouts can indeed enable rabbits as an immunocompetent animal model for AIDS.

**TABLE 2:** HIV-1 infection and replication barriers in mice, rats, and rabbits*.

HIV-1 replication steps	Human	Mouse	Rat	Rabbit	Key genes to be targeted in rabbits
1. Virus entry	Y	X	X	X	CD4, CCR5
2. Reverse transcription	Y	Y	Y	X	TRIM5
3. Integration	Y	Y	Y	Y	
4. Transcription	Y	X	X	Y	
5. vRNA export	Y	X	X	Y	
6. Assembly and egress	Y	X	Y in macrophages X in T-cell	Y	
7. Infectivity	Y	X	X	Y in T-cells X in macrophages	APOBEC1
Overall efficiency	★★★★★	★★	★★	★★★★	

*adapted from ref (Tervo and Keppler, 2010). Y, high efficiency; X, low efficiency. ★: efficiency indicator, with five stars the most, and one star the least efficient.

## Gene Edited Rabbits for Translational Studies of Cystic Fibrosis

Cystic fibrosis (CF) is the most common fatal autosomal recessive disorder with a disease frequency of one in 2,000 live births and a carrier rate of approximately 5% in the Caucasian population (Pilewski and Frizzell, 1999). The disease can be characterized as a malfunction of exocrine tissues in which the abnormal regulation of epithelial chloride channels is associated with disease pathophysiology (Welsh and Ramsey, 1998). The major clinical symptoms include chronic pulmonary disease, pancreatic exocrine insufficiency, intestinal disease and an increase in the concentration of sweat chloride (Wang et al., 2014).

Mutations of the gene encoding cystic fibrosis transmembrane conductance regulator (CFTR) lead to CF (Wang et al., 2014). The CFTR protein is a multi-domain integral membrane glycoprotein, which functions as a regulator of chloride and other ion transport across the cell membrane. More than 2,000 CF mutations have been identified in the CFTR gene. The most common mutation in CF is the deletion of the phenylalanine residue at position 508 (ΔF508), which occurs in more than 70% of homozygous CF patients. Accordingly, the majority of research on CF has been focused on ΔF508.

CF mouse models have made significant contributions toward our understanding of the disease and the development of therapies (Grubb and Boucher, 1999; Keiser and Engelhardt, 2011; Wang et al., 2014). Various CF mouse models have been developed, such as the exon 10 “knockout” models (Colledge et al., 1992; Snouwaert et al., 1992), the ΔF508 models (Colledge et al., 1995; van Doorninck et al., 1995; Zeiher et al., 1995), and the G551D models (Delaney et al., 1996). However, it is recognized that there are significant limitations in translating the information gained from CF mice to human patients. For example, unlike human CF patients, CF mice show a lack of pulmonary pathophysiology, do not exhibit obvious pancreatic pathology, and have no male infertility problems. Such differences demonstrate the need for alternative animal models for CF research.

CFTR KO ferrets (Sun et al., 2010), and CFTR KO and ΔF508 pigs (Rogers et al., 2008a,b), were generated by nuclear transfer (NT). These large animal models have been shown to more closely mimic conditions observed in human CF patients, including the lung, pancreatic and liver phenotypes that were not often found in CF mice. However, neither pig nor ferret is a convenient laboratory species. Both are associated with high maintenance costs and require specialized handling skills and husbandry infrastructure. These factors have limited the applicability of CF pigs and ferrets almost exclusively to the labs originally produced these animals and a few closely associated with them.

In 2020, Xu et al. (2020) reported the production of CF rabbit models by CRISPR/Cas9. CF rabbits manifest many typical disease phenotypes, including and beyond those observed in CF mice. Gut obstruction, pancreatic, and liver destruction are clearly seen in CF rabbits and are comparable to the human condition. CF rabbits exhibited human CF-like abnormalities in the bioelectric properties of the nasal and tracheal epithelia. Some, albert a small percentage, CF rabbits were found with spontaneous bacterial infections in the airways. Furthermore, the CF rabbits have a relatively long lifespan. Without expensive treatments and surgery to correct the meconium ileus defects, CF rabbits survive on average 80 days. The life span is longer than that of CF pigs and ferrets which have required major surgical procedures in order to sustain life past the first few couple weeks, and will provide researchers a clinically relevant timeframe to delineate the pathogenesis of the CF. Future genetic manipulations may make these CF rabbit models even more useful, including gut-specific expression of CFTR or lung-specific knockout of CFTR. Modifications such as these would minimize the development of debilitating intestinal obstruction and would allow for the clinical development of CF airway disease models as well as other CF complications which are urgently needed in CF translational studies.

## Gene Edited Rabbits for Translational Studies of Ocular Diseases

Globally, at least 2.2 billion people have a vision impairment and of these, over 100 million individuals are affected by irreversible visual impairments and blindness. Development of novel ocular disease therapeutics requires animal models capable of developing ocular diseases with similar etiology, pathology and suitability for future trials of new therapeutic approaches. While most experimental ophthalmology and visual research is performed on rodent models, these animals are often unsuitable for pre-clinical drug efficacy and safety studies, due to significant differences in pharmacokinetics and the required volumes and dosages, when compared to humans. In addition, the small eyeball of mice and rats makes it hard to physically access for clinical manipulations and thus limit the biomedical applications of these animal models of ocular diseases.

Rabbit models of ocular diseases are particularly useful in this context, especially considering that substantial knowledge already exists regarding the retinal circuitry, anatomy, and ophthalmologic pathogenesis in rabbits (Reichenbach et al., 1991a,b,c, 1993, 1994; Jones et al., 2011; Abdo et al., 2017). Rabbits possess relatively large eyes that share many anatomical features with humans. These similarities include eyeball size, internal eyeball structure, eyeball optical system, eyeball biomechanics, and eyeball biochemical features (Zhou et al., 2008). Because of these similarities, various physiologic manipulation technologies and equipment developed for human eyes, for both invasive (vitrectomy and subretinal injections) and non-invasive interventions, can be performed in rabbits with minimal modification. Rabbits also have a relatively long lifespan, enabling studies of age-related aspects of retinal degenerative diseases and long-term assessment of therapeutic and side effects (Comfort, 1959). Rabbits have been used successfully as models for clinical, morphological and mechanistic studies of both common and rare ocular diseases, including DES, glaucoma, AMD, light-induced retinopathies, cataracts and uveitis, diabetic retinopathy, retinal detachment and proliferative vitreoretinopathy, ocular allergy, retinoblastoma, and retinitis pigmentosa (Hasumura et al., 2000; Kondo et al., 2009; Jones et al., 2011; Del Amo and Urtti, 2015; Ahn et al., 2016; Zernii et al., 2016). However, most of these rabbit models were induced experimentally, which limited their application in hereditary eye diseases. Due to the lack of a technology to generate targeted gene modifications in rabbits, for many years, genetically modified rabbit models for ocular disease studies were restricted to transgenic rabbits only (Kondo et al., 2009; Yokoyama et al., 2010; Hirota et al., 2012; Ueno et al., 2013, 2019; Asakawa et al., 2015, 2016; Nagai et al., 2016; Nakagami et al., 2016; Kominami et al., 2017, 2019; Okado et al., 2017; Roy et al., 2019).

Recently, the extension of gene targeting to rabbits with CRISPR/Cas9 technology has motivated efforts to develop gene targeted rabbit models that replicate inherited human eye diseases. Cataract development is the largest contributor to global blindness in adults aged 50 years and older, and causes approximately 45% of global blindness (GBD 2019 Blindness and Vision Impairment Collaborators, 2020). In 2016, Lai group firstly reported that GJA8 gene knockout rabbits replicated the congenital cataracts phenotype (Yuan et al., 2016). In 2017, the same group reported another rabbit model of congenital cataracts by αA-crystallin gene knockout, which recapitulates phenotypes of congenital cataracts, microphthalmia, obscurity, and early atrophy of the lens, and failed differentiation of lens fibers (Yuan et al., 2017).

These achievements suggest the possibility of establishing GE rabbit models for the study of other ocular diseases. For example, Usher syndrome (USH) is a genetic disorder resulting in a combination of hearing loss, visual impairment and, in some types, balance issues, and currently has no effective treatment (Mathur and Yang, 2015). The major ocular symptoms of USH patients is retinitis pigmentosa caused photoreceptor degeneration in the retina. At least 10 mouse models of USH have been developed with the hearing defects resembling those of human patients. Interestingly, these mouse models with mutant USH genes typically have no or weak retinal phenotype (Liu et al., 2007; Lefèvre et al., 2008; Lentz et al., 2010; Alagramam et al., 2011; Mathur and Yang, 2015). An alternative USH animal model with eye phenotypes hence will be of great translational value. In this regard, gene editing tools such as CRISPR/Cas9 may facilitate the development of USH rabbit models with the hope that these animals will replicate the major retinal degeneration features that were not observed in mouse models. It is important here to point out that ocular diseases are in general are prime candidates be treated with gene editing therapies, due to reasons such as the easy physical access to the eyes, the immune privilege status of the eyes, and the luxury of treating one eye at a time. The application of genome editing technology to establish a rabbit model of ocular hereditary diseases therefore would not only provide some clinically relevant animal models but also serve as a preclinical model system to develop and validate gene editing strategies.

## Gene Edited Rabbits for Translational Studies of Muscular Dystrophy

Muscular dystrophies are a large group of genetically inherited degenerative muscle disorders, sharing clinical features of progressive muscle weakness, and dystrophic pathological appearance on muscle biopsy. These muscle diseases are caused by mutations in more than 60 genes (Benarroch et al., 2019). A number of naturally occurring and GE mammalian animal models have been established to study the pathophysiological mechanisms and develop therapeutic treatments for various types of muscular dystrophies, including mice, rats, dogs and pigs. The rabbit model is a new addition to the list. Although no animal model is perfect for every purpose, each has contributed to the pathogenesis characterization and preclinical studies.

The *mdx* mice, the most commonly used mouse model of Duchenne muscular dystrophy (DMD) for decades, develop the muscular dystrophy phenotype, but their clinical features are much milder when compared to DMD patients. These mice are larger in size than their wild-type littermates, live about 75% of normal lifespan, and do not present overt cardiomyopathy. Sui et al. (2018a) reported a rabbit model of DMD in 2018. Using zygote microinjection of CRISPR-Cas9 mRNA and a guide RNA designed to target exon 51, Sui et al. established a DMD rabbit with disrupted expression of dystrophin. The DMD rabbits exhibit the typical muscular dystrophy phenotypes including elevated serum creatine kinase (CK) levels, muscle necrosis, regeneration, central nucleation, fiber size variation, fibrosis, and fatty replacement. The forelimb of DMD rabbits exhibited paralysis. The physical activity of DMD rabbits was significantly impaired as examined by a 1-h wearable device-assisted open field test. These rabbits also failed to climb up a stair-step. Premature death occurs with 50% morbidity by around 6 months. Similar to DMD patients, cardiomyopathy was readily detectable beginning at 5 months of age. Echocardiography recording showed that the DMD rabbits had chamber dilation with decreased ejection fraction and fraction shortening. As compared with the *mdx* mice, the DMD rabbits appeared to more faithfully recapitulate the DMD pathology and could be useful for the translational studies.

The *mdx* mouse is not a unique case in which the disease course does not resemble their human counterpart well in muscular dystrophy. Previously, Xu et al. (2015) reported that genetic disruption of Ano5 in mice did not lead to the development of muscular dystrophy, in contrast to the reports that mutations in *ANO5* are responsible for limb girdle muscular dystrophy type 2L (LGMD2L) or Miyoshi myopathy type 3 (MMD3) in patients (Bolduc et al., 2010; Hicks et al., 2011; Magri et al., 2012; Pénisson-Besnier et al., 2012; Schessl et al., 2012; Little et al., 2013; Witting et al., 2013). Sui et al. (2018b) took a similar approach as for DMD rabbits to generate an Ano5-mutant rabbit model with CRISPR-engineered small indels in the exon 12 and/or 13 of *ANO5* gene. The Ano5-mutant rabbits developed clear signs of muscular dystrophy with increased serum CK levels, muscle necrosis, regeneration, fatty replacement, and fibrosis starting from 12 months of age.

Considering the short gestational period, high prolificness and the relatively inexpensive maintenance cost as compared to large animal models such as dogs and pigs, the rabbit models provide an excellent alternative for muscular dystrophy research.

## Conclusion

In this review, we presented several examples where GE rabbits may find unique applications in translational biomedical research. Needless to say, these only represent a subset of the potential uses of these models. For example, several immunodeficient rabbit lines have been produced by knocking out of genes such as Il2rg, Foxn1, and Rag2 (Song et al., 2013, 2017; Yan et al., 2014; Hashikawa et al., 2020); these animals are expected to make valuable contributions to regenerative medicine, cancer and primary immunodeficiency, as comprehensively reviewed elsewhere (Song et al., 2020). On the other hand, we want to point out that no model system is perfect. Just as we have shown many times where mouse models are often suboptimal, rabbit models are not silver bullets for modeling human diseases either. Researchers should choose a model animal primarily based on the scientific questions to be answered. With this in mind, current and future GE rabbit models are expected to find unique opportunities to facilitate translational biomedical research.

## Author Contributions

JX, JZ, RH, and YC conceived the topic. JX, JZ, DY, JS, BP, CZ, JH, XP, NC, RH, and YC wrote the manuscript. All authors contributed to the article and approved the submitted version.

## Conflict of Interest

The authors declare that the research was conducted in the absence of any commercial or financial relationships that could be construed as a potential conflict of interest.
