# Supplementary material for: Gene Editing in Rabbits: Unique Opportunities for Translational Biomedical Research
Source: Front Genet. 2021 Jan 28;12:642444. doi: 10.3389/fgene.2021.642444 (PMC7876448; doi:10.3389/fgene.2021.642444)
Supplement: Supplementary file 1 [file Table_1.DOCX]

**Title**:

Gene editing in rabbits: unique opportunities for translational biomedical research

**Supplementary information**

**Supplementary Table 1**. List of GE rabbit publications (retrieved from pubmed.gov on Dec 7 2020). Z: ZFN. T: TALEN. C: CRISPR/Cas. P: platform. G: genetic disease. V: cardiovascular disease. E: eye disease. O: others. Please refer to references for full gene name(s).

| Seq | Gene targeted | Nuclease used | | | Disease modeled/potential application |  |  |  |  |  | Ref | Year |
| --- | --- | --- | --- | --- | --- | --- | --- | --- | --- | --- | --- | --- |
|  |  | Z | T | C |  | P | G | V | E | O |  |  |
| 1 | IgM | x |  |  | Production of human antibodies in rabbits |  |  |  |  | x | (1) | 2011 |
| 2 | Rag1 and Rag2 |  | x |  | Primary immunodeficiency |  | x |  |  |  | (2) | 2013 |
| 3 | ApoC3 | x |  |  | CVD |  |  | x |  |  | (3) | 2013 |
| 4 | Multiple genes |  | x |  | Establish gene editing platform in rabbits | x |  |  |  |  | (4) | 2014 |
| 5 | TYR |  |  | x | Albinism |  | x |  |  |  | (5) | 2014 |
| 6 | ApoE | x |  |  | CVD |  |  | x |  |  | (6) | 2014 |
| 7 | Multiple genes |  |  | x | Establish multiplex gene editing platform in rabbits | x |  |  |  |  | (7) | 2014 |
| 8 | TYR |  |  | x | Albinism |  | x |  |  |  | (8) | 2016 |
| 9 | Multiple genes |  |  | x | Improve gene editing platform in rabbits | x |  |  |  |  | (9) | 2016 |
| 10 | GJA8 |  |  | x | Eye disease |  |  |  | x |  | (10) | 2016 |
| 11 | MSTN |  |  | x | Improve muscle growth |  |  |  |  | x | (11) | 2016 |
| 12 | Rosa26 |  |  | x | Establish safe harbor knock-in in rabbits | x |  |  |  |  | (12) | 2016 |
| 13 | PHEX |  |  | x | X-linked hypophosphatemia |  | x |  |  |  | (13) | 2016 |
| 14 | Fumarylacetoacetate Hydrolase |  |  | x | Hereditary Tyrosinemia  Type 1 |  | x |  |  |  | (14) | 2017 |
| 15 | CETP | x |  |  | CVD |  |  | x |  |  | (15) | 2017 |
| 16 | αA-Crystallin |  |  | x | Eye disease |  |  |  | x |  | (16) | 2017 |
| 17 | TYR |  |  | x | Albinism | x |  |  |  |  | (17) | 2017 |
| 18 | SRY |  |  | x | Sex reversal |  |  |  |  | x | (18) | 2017 |
| 19 | Foxn1, Rag2, Il2rg, Prkdc |  |  | x | Primary immunodeficiency |  | x |  |  |  | (19) | 2017 |
| 20 | FUT1, FUT2 and SEC1 |  |  | x | Multiplex knockout of homologous genes | x |  |  |  |  | (20) | 2018 |
| 21 | ATP7B |  |  | x | Wilson disease |  | x |  |  |  | (21) | 2018 |
| 22 | SRY |  |  | x | Hermaphroditism |  | x |  |  |  | (22) | 2018 |
| 23 | fibrillin-1 |  |  | x | Marfanoid-progeroid-lipodystrophy  syndrome |  | x |  |  |  | (23) | 2018 |
| 24 | Multiple genes |  |  | x | Establish Cas12a gene editing platform in rabbits | x |  |  |  |  | (24) | 2018 |
| 25 | ANO5 |  |  | x | muscular dystrophy |  | x |  |  |  | (25) | 2018 |
| 26 | Dystrophin |  |  | x | Duchenne muscular dystrophy |  | x |  |  |  | (26) | 2018 |
| 27 | Multiple genes |  |  | x | Establish base editing platform in rabbits | x |  |  |  |  | (27) | 2018 |
| 28 | Paired-homeodomain transcription factor 4 (PAX4) |  |  | x | Diabetes |  |  |  |  | x | (28) | 2018 |
| 29 | LDLR |  |  | x | CVD |  |  | x |  |  | (29) | 2018 |
| 30 | TYR |  |  | x | Albinism |  | x |  |  |  | (30) | 2018 |
| 31 | CLPG1 |  |  | x | muscular hypertrophy  syndrome |  | x |  |  |  | (31) | 2019 |
| 32 | LMNA |  |  | x | Premature Aging Syndrome |  | x |  |  |  | (32) | 2019 |
| 33 | DMP1 |  |  | x | autosomal recessive form of hypophosphatemic rickets (ARHR) |  | x |  |  |  | (33) | 2019 |
| 34 | ApoE and LDLR |  |  | x | CVD |  |  | x |  |  | (34) | 2019 |
| 35 | Multiple genes |  |  | x | Improve Cas9 base editor in rabbits | x |  |  |  |  | (35) | 2019 |
| 36 | Myostatin |  |  | x | Improve muscle growth |  |  |  |  | x | (36) | 2019 |
| 37 | GADD45G |  |  | x | cleft lip with or without cleft palate (CL/P) |  | x |  |  |  | (37) | 2019 |
| 38 | β-casein (CSN2) |  |  | x | Safe harbor knock-in of a transgene |  |  |  |  | x | (38) | 2019 |
| 39 | MC1R |  |  | x | Coat color change |  |  |  |  | x | (39) | 2019 |
| 40 | glucokinase |  |  | x | Diabetes |  |  |  |  | x | (40) | 2019 |
| 41 | Multiple genes |  |  | x | Improve base editing in rabbits | x |  |  |  |  | (41) | 2019 |
| 42 | Multiple genes |  |  | x | Improve base editing in rabbits | x |  |  |  |  | (42) | 2020 |
| 43 | Fibroblast Growth Factor 5 |  |  | x | Increase hair length |  |  |  |  | x | (43) | 2020 |
| 44 | Connective  tissue growth factor |  |  | x | Eye disease |  |  |  | x |  | (44) | 2020 |
| 45 | Il2rg |  |  | x | Primary immunodeficiency |  | x |  |  |  | (45) | 2020 |
| 46 | Otc and Fgf5 |  |  | x | Using base editor to target start codon to achieve knockout | x |  |  |  |  | (46) | 2020 |
| 47 | ApoC3 | x |  |  | CVD |  |  | x |  |  | (47) | 2020 |
| 48 | Multiple genes |  |  | x | Improve base editing in rabbits | x |  |  |  |  | (48) | 2020 |
| 49 | cystathionine  β-synthase |  |  | x | Congenital hyper-homocysteinemia |  | x |  |  |  | (49) | 2020 |
| 50 | Fumarylacetoacetate Hydrolase |  |  | x | To correct hereditary  tyrosinemia type I |  | x |  |  |  | (50) | 2020 |
| 51 | CFTR |  |  | x | Cystic fibrosis |  | x |  |  |  | (51) | 2020 |
| 52 | WAS |  |  | x | Wiskott-Aldrich syndrome |  | x |  |  |  | (52) | 2020 |

**Reference for Supplementary Data**

1. T. Flisikowska *et al.*, Efficient immunoglobulin gene disruption and targeted replacement in rabbit using zinc finger nucleases. *PLoS One* **6**, e21045 (2011).

2. J. Song *et al.*, Generation of RAG 1- and 2-deficient rabbits by embryo microinjection of TALENs. *Cell Res* **23**, 1059-1062 (2013).

3. D. Yang *et al.*, Production of apolipoprotein C-III knockout rabbits using zinc finger nucleases. *J Vis Exp* 10.3791/50957, e50957 (2013).

4. Y. Wang *et al.*, Generation of knockout rabbits using transcription activator-like effector nucleases. *Cell Regen* **3**, 3 (2014).

5. A. Honda *et al.*, Single-step generation of rabbits carrying a targeted allele of the tyrosinase gene using CRISPR/Cas9. *Exp Anim* **64**, 31-37 (2015).

6. D. Ji, G. Zhao, A. Songstad, X. Cui, E. J. Weinstein, Efficient creation of an APOE knockout rabbit. *Transgenic Res* **24**, 227-235 (2015).

7. Q. Yan *et al.*, Generation of multi-gene knockout rabbits using the Cas9/gRNA system. *Cell Regen* **3**, 12 (2014).

8. Y. Song *et al.*, Efficient dual sgRNA-directed large gene deletion in rabbit with CRISPR/Cas9 system. *Cell Mol Life Sci* **73**, 2959-2968 (2016).

9. J. Song *et al.*, RS-1 enhances CRISPR/Cas9- and TALEN-mediated knock-in efficiency. *Nat Commun* **7**, 10548 (2016).

10. L. Yuan *et al.*, CRISPR/Cas9-mediated GJA8 knockout in rabbits recapitulates human congenital cataracts. *Sci Rep* **6**, 22024 (2016).

11. Q. Lv *et al.*, Efficient Generation of Myostatin Gene Mutated Rabbit by CRISPR/Cas9. *Sci Rep* **6**, 25029 (2016).

12. D. Yang *et al.*, Identification and characterization of rabbit ROSA26 for gene knock-in and stable reporter gene expression. *Sci Rep* **6**, 25161 (2016).

13. T. Sui *et al.*, CRISPR/Cas9-mediated mutation of PHEX in rabbit recapitulates human X-linked hypophosphatemia (XLH). *Hum Mol Genet* **25**, 2661-2671 (2016).

14. L. Li *et al.*, Fumarylacetoacetate Hydrolase Knock-out Rabbit Model for Hereditary Tyrosinemia Type 1. *J Biol Chem* **292**, 4755-4763 (2017).

15. J. Zhang *et al.*, Deficiency of Cholesteryl Ester Transfer Protein Protects Against Atherosclerosis in Rabbits. *Arterioscler Thromb Vasc Biol* **37**, 1068-1075 (2017).

16. L. Yuan *et al.*, CRISPR/Cas9-Mediated Mutation of alphaA-Crystallin Gene Induces Congenital Cataracts in Rabbits. *Invest Ophthalmol Vis Sci* **58**, BIO34-BIO41 (2017).

17. Y. Song *et al.*, CRISPR/Cas9-mediated mutation of tyrosinase (Tyr) 3' UTR induce graying in rabbit. *Sci Rep* **7**, 1569 (2017).

18. Y. Song *et al.*, Mutation of the Sp1 binding site in the 5' flanking region of SRY causes sex reversal in rabbits. *Oncotarget* **8**, 38176-38183 (2017).

19. J. Song *et al.*, Production of immunodeficient rabbits by multiplex embryo transfer and multiplex gene targeting. *Sci Rep* **7**, 12202 (2017).

20. H. Liu *et al.*, Multiple homologous genes knockout (KO) by CRISPR/Cas9 system in rabbit. *Gene* **647**, 261-267 (2018).

21. W. Jiang *et al.*, Production of Wilson Disease Model Rabbits with Homology-Directed Precision Point Mutations in the ATP7B Gene Using the CRISPR/Cas9 System. *Sci Rep* **8**, 1332 (2018).

22. Y. Song *et al.*, CRISPR/Cas9-mediated mosaic mutation of SRY gene induces hermaphroditism in rabbits. *Biosci Rep* **38** (2018).

23. M. Chen *et al.*, Truncated C-terminus of fibrillin-1 induces Marfanoid-progeroid-lipodystrophy (MPL) syndrome in rabbit. *Dis Model Mech* **11** (2018).

24. H. Wu *et al.*, Engineering CRISPR/Cpf1 with tRNA promotes genome editing capability in mammalian systems. *Cell Mol Life Sci* **75**, 3593-3607 (2018).

25. T. Sui *et al.*, Development of muscular dystrophy in a CRISPR-engineered mutant rabbit model with frame-disrupting ANO5 mutations. *Cell Death Dis* **9**, 609 (2018).

26. T. Sui *et al.*, A novel rabbit model of Duchenne muscular dystrophy generated by CRISPR/Cas9. *Dis Model Mech* **11** (2018).

27. Z. Liu *et al.*, Highly efficient RNA-guided base editing in rabbit. *Nat Commun* **9**, 2717 (2018).

28. Y. Xu *et al.*, Generation and Phenotype Identification of PAX4 Gene Knockout Rabbit by CRISPR/Cas9 System. *G3 (Bethesda)* **8**, 2833-2840 (2018).

29. R. Lu *et al.*, Spontaneous severe hypercholesterolemia and atherosclerosis lesions in rabbits with deficiency of low-density lipoprotein receptor (LDLR) on exon 7. *EBioMedicine* **36**, 29-38 (2018).

30. Y. Song *et al.*, Functional validation of the albinism-associated tyrosinase T373K SNP by CRISPR/Cas9-mediated homology-directed repair (HDR) in rabbits. *EBioMedicine* **36**, 517-525 (2018).

31. Y. Wan *et al.*, Efficient generation of CLPG1-edited rabbits using the CRISPR/Cas9 system. *Reprod Domest Anim* **54**, 538-544 (2019).

32. T. Sui *et al.*, LMNA-mutated Rabbits: A Model of Premature Aging Syndrome with Muscular Dystrophy and Dilated Cardiomyopathy. *Aging Dis* **10**, 102-115 (2019).

33. T. Liu *et al.*, DMP1 Ablation in the Rabbit Results in Mineralization Defects and Abnormalities in Haversian Canal/Osteon Microarchitecture. *J Bone Miner Res* **34**, 1115-1128 (2019).

34. T. Yuan *et al.*, Generation of hyperlipidemic rabbit models using multiple sgRNAs targeted CRISPR/Cas9 gene editing system. *Lipids Health Dis* **18**, 69 (2019).

35. Z. Liu *et al.*, Improved base editor for efficient editing in GC contexts in rabbits with an optimized AID-Cas9 fusion. *FASEB J* **33**, 9210-9219 (2019).

36. T. Zhang *et al.*, 'Double-muscling' and pelvic tilt phenomena in rabbits with the cystine-knot motif deficiency of myostatin on exon 3. *Biosci Rep* **39** (2019).

37. Y. Lu *et al.*, Mutations of GADD45G in rabbits cause cleft lip by the disorder of proliferation, apoptosis and epithelial-mesenchymal transition (EMT). *Biochim Biophys Acta Mol Basis Dis* **1865**, 2356-2367 (2019).

38. H. Li *et al.*, Site-specific integration of rotavirus VP6 gene in rabbit beta-casein locus by CRISPR/Cas9 system. *In Vitro Cell Dev Biol Anim* **55**, 586-597 (2019).

39. N. Xiao *et al.*, A Novel Pale-Yellow Coat Color of Rabbits Generated via MC1R Mutation With CRISPR/Cas9 System. *Front Genet* **10**, 875 (2019).

40. Y. Song *et al.*, Genetic deletion of a short fragment of glucokinase in rabbit by CRISPR/Cas9 leading to hyperglycemia and other typical features seen in MODY-2. *Cell Mol Life Sci* **77**, 3265-3277 (2020).

41. Z. Liu *et al.*, Highly efficient base editing with expanded targeting scope using SpCas9-NG in rabbits. *FASEB J* **34**, 588-596 (2020).

42. Z. Liu *et al.*, Efficient base editing with high precision in rabbits using YFE-BE4max. *Cell Death Dis* **11**, 36 (2020).

43. Y. Xu *et al.*, CRISPR/Cas9-mediated Disruption of Fibroblast Growth Factor 5 in Rabbits Results in a Systemic Long Hair Phenotype by Prolonging Anagen. *Genes (Basel)* **11** (2020).

44. E. J. Lee, J. C. Han, D. Y. Park, J. Cho, C. Kee, Effect of connective tissue growth factor gene editing using adeno-associated virus-mediated CRISPR-Cas9 on rabbit glaucoma filtering surgery outcomes. *Gene Ther* 10.1038/s41434-020-0166-4 (2020).

45. Y. Hashikawa *et al.*, Generation of knockout rabbits with X-linked severe combined immunodeficiency (X-SCID) using CRISPR/Cas9. *Sci Rep* **10**, 9957 (2020).

46. S. Chen *et al.*, CRISPR Start-Loss: A Novel and Practical Alternative for Gene Silencing through Base-Editing-Induced Start Codon Mutations. *Mol Ther Nucleic Acids* **21**, 1062-1073 (2020).

47. H. Yan *et al.*, Apolipoprotein CIII Deficiency Protects Against Atherosclerosis in Knockout Rabbits. *Arterioscler Thromb Vasc Biol* **40**, 2095-2107 (2020).

48. Z. Liu *et al.*, Precise base editing with CC context-specificity using engineered human APOBEC3G-nCas9 fusions. *BMC Biol* **18**, 111 (2020).

49. T. Zhang *et al.*, Hyperhomocysteinemia and dyslipidemia in point mutation G307S of cystathionine beta-synthase-deficient rabbit generated using CRISPR/Cas9. *Lipids Health Dis* **19**, 224 (2020).

50. N. Li *et al.*, CRISPR/Cas9-Mediated Gene Correction in Newborn Rabbits with Hereditary Tyrosinemia Type I. *Mol Ther* 10.1016/j.ymthe.2020.11.023 (2020).

51. J. Xu *et al.*, Phenotypes of CF rabbits generated by CRISPR/Cas9-mediated disruption of the CFTR gene. *JCI Insight* 10.1172/jci.insight.139813 (2020).

52. J. Zhou *et al.*, Development of a rabbit model of Wiskott-Aldrich syndrome. *FASEB J* 10.1096/fj.202002118RR (2020).
